# Antidurotaxis Droplet Motion onto Gradient Brush Substrates

**DOI:** 10.1021/acs.langmuir.3c01999

**Published:** 2023-09-06

**Authors:** Russell Kajouri, Panagiotis E. Theodorakis, Jan Židek, Andrey Milchev

**Affiliations:** † Institute of Physics, Polish Academy of Sciences, Al. Lotników 32/46, 02-668 Warsaw, Poland; ‡ Central European Institute of Technology, Brno University of Technology, Purkyňova 656/123, 612 00 Brno, Czech Republic; ¶ Bulgarian Academy of Sciences, Institute of Physical Chemistry, 1113 Sofia, Bulgaria

## Abstract

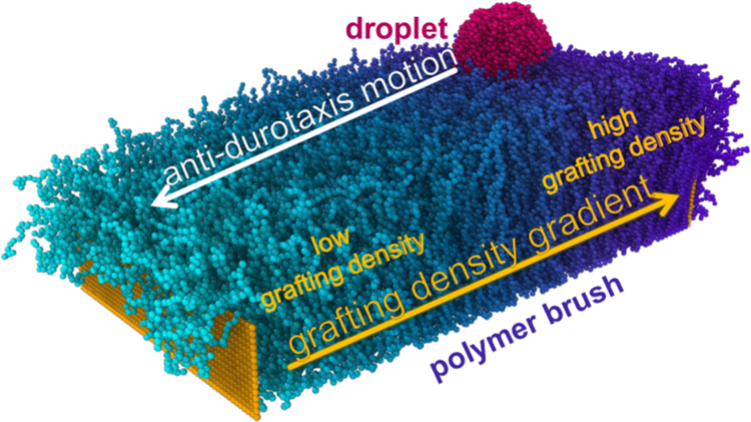

Durotaxis motion is a spectacular phenomenon manifesting itself by the autonomous motion of a nano-object between parts of a substrate with different stiffness. This motion usually takes place along a stiffness gradient from softer to stiffer parts of the substrate. Here, we propose a new design of a polymer brush substrate that demonstrates antidurotaxis droplet motion, that is, droplet motion from stiffer to softer parts of the substrate. By carrying out extensive molecular dynamics simulation of a coarse-grained model, we find that antidurotaxis is solely controlled by the gradient in the grafting density of the brush and is favorable for fluids with a strong attraction to the substrate (low surface energy). The driving force of the antidurotaxial motion is the minimization of the droplet–substrate interfacial energy, which is attributed to the penetration of the droplet into the brush. Thus, we anticipate that the proposed substrate design offers a new understanding and possibilities in the area of autonomous motion of droplets for applications in microfluidics, energy conservation, and biology.

## Introduction

The spontaneous motion of nano-objects onto substrates has attracted much interest due to its potential impact in applications, such as microfluidics, microfabrication, coatings, energy conversion, and biology.^1−12^ In this kind of autonomous motion, the direction of motion can also be controlled and steered by gradient changes in a substrate property that can be “sensed” by the nano-object. More specifically, such a property can be the stiffness of the substrate, which can enable and sustain the motion of the nano-object in a specific direction. A characteristic example here is the motion of cells on tissues, known as durotaxis.^11−15^ Apart from biological systems, however, durotaxis has also been realized in the case of a spectrum of different nano-objects (e.g., fluids), both in theoretical and simulation models,^16−23^ as well as in experiments.^24^

An important aspect of the durotaxis motion is that the nano-object can sustain the motion without external energy supply. However, apart from stiffness gradients, such motion can also be caused by specific substrate patterns. Characteristic examples here are rugotaxis, where the motion of fluids is provoked by a gradient in the wavelength that characterizes wavy substrates^25,26^ and curvotaxis driven by curved protein complexes at the cell.^27^ Other possibilities may include the transport of small condensate droplets on asymmetric pillars,^28^ three-dimensional capillary ratchets,^29^ or taking advantage of pinning and depinning effects at the three-phase contact line.^30^ Moreover, in the case of capillary ratchets, the motion can take place along or against the gradient depending on the surface tension of the fluid.^29^ Wettability gradients have also been exploited to steer the motion of fluids,^31−33^ while the long-range transport of fluids can be realized by using electrostatic^34,35^ or triboelectric charges.^36^ In contrast, the use of external fields, such as in the case of electrotaxis,^37^ requires energy supply by an external source, as, also, in the case of thermotaxis to sustain a temperature gradient.^38^ Other options requiring external sources may include the use of electrical current,^39−42^ charge,^43−45^ or even simple stretching,^46^ as well as chemically driven droplets,^47,48^ droplets on vibrated substrates^49−52^ or wettability ratchets.^53−56^

In our previous studies, we have investigated various substrate designs that can cause and sustain the motion of liquid droplets,^16,23,25^ which were mainly motivated by relevant experiments.^24,26^ In particular, in the context of durotaxis droplet motion, a new design based on brush polymer substrates was proposed, where the stiffness gradient was imposed by varying the chain stiffness of the grafted polymers along the gradient for a given grafting density.^23^ In this case, it has been found that the grafting density of polymer chains and the droplet adhesion to the brush are the key parameters determining whether the motion will be realized, as well as its efficiency. In particular, it has been found that moderate values of both will promote droplet motion. Surprisingly, the stiffness gradient itself, albeit necessary for the durotaxis motion, turned out to be irrelevant for determining the efficiency of the motion in terms of the average velocity of the droplet. Importantly, the direction of the droplet motion was in the same direction as the stiffness gradient, i.e., from softer to stiffer parts of the substrate. In effect, this translates into varying substrate roughness, which drives the droplet motion. In contrast, in a specific experiment,^24^ the direction of motion of droplets on a soft, silicon-gel substrate has been from the stiffer toward the softer parts of the substrate for μm scale droplets, which is well below the capillary length scale (∼2.5 mm) in the case of water droplets. Although for this reason gravity seems not to play an important role, durotaxis has been more efficient in the case of larger droplets.^24^ While biological systems^11−15^ and simulation models^16,17,20,23^ have been thus far only able to demonstrate the droplet motion in the direction of the stiffness gradient, that is from softer to stiffer parts of a substrate, to the best of our knowledge, there is currently no *in silico* substrate design that has demonstrated droplet motion in the opposite direction of the stiffness gradient, namely, from the stiffer toward the softer parts of the substrate.

Motivated by relevant experiments^24^ and previous experience with gradient brush substrates,^23^ we consider a polymer brush that can initiate and sustain the droplet motion toward the softer parts of the substrate. Here, we will refer to this kind of motion as “antidurotaxis” in order to underline the fact that the droplet moves in the opposite direction with respect to a positive stiffness gradient. In this new design of the brush substrate, the stiffness gradient is implemented by the gradual change in the grafting density of fully flexible polymer chains. By using extensive molecular dynamics (MD) simulations of a coarse-grained (CG) model, we explore the key parameters of the system such as the gradient in the grafting density, the droplet attraction to the substrate, the droplet size, and the viscosity. Our method also provides the molecular-scale resolution required to explore the underlying mechanism of the antidurotaxis motion. In this way, our study casts further light on the self-sustained motion of droplets onto brush gradient substrates, and, as a result, unravels new possibilities in nanoscale science and technology^19^ for various medicine and engineering applications.^12,57^ Moreover, brush substrates share structural characteristics with various biological surfaces that can expel various exogenous substances from their structure,^58^ such as the mucus layer from airway epithelia.^59^ Also, in the context of regenerative medicine,^12^ the concept of gradient substrates plays an important role for applications in this area, for example, in drug transport within the body. Hence, we anticipate that our study will have a broader impact beyond engineering applications. Moreover, the fact that the design of the brush substrate only depends on the variation (gradient) of the grafting density, compared to a previous design that the chain stiffness and possibly the chemistry of the chains had to be varied to tune the substrate properties,^23^ might suggest that the brush system investigated here might hold greater hope for experiments, thus offering further possibilities for relevant applications. In the following, we provide details of the system, simulation model, and methodology. Then, we will present and discuss the obtained results, while we will draw the conclusions resulting from our investigations in the final section.

## Materials and Methods

**Figure 1:**
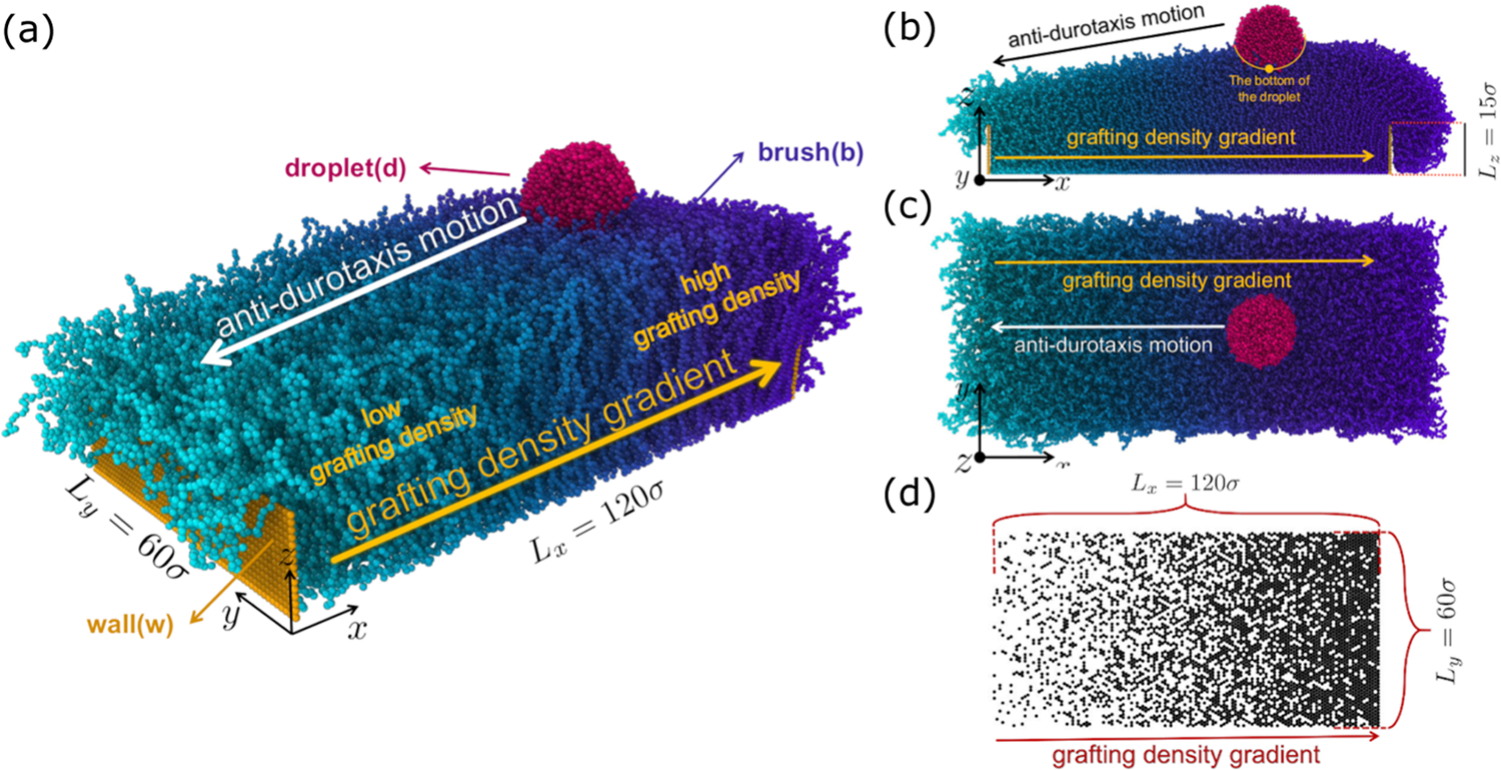
(a) Typical initial configuration of the system. The droplet is placed on the substrate side with the highest grafting density, which is here σ_g,h_ = 0.6 σ^–2^, while on the other side, the lowest grafting density is σ_g,l_ = 0.1 σ^–2^. The length of the system in the gradient direction, *x*, is *L*_*x*_ = 120 σ, with the grafting density gradient defined as *G* = (σ_g,h_–σ_g,l_) /*L*_*x*_. Droplet and substrate polymer chains consist of fully flexible chains of length *N*_d_ = 10 and *N*_b_ = 50 beads, respectively, while the total size of the droplet is *N* = 4000 beads in this case. Also, the strength of the interaction between the droplet and substrate beads is ε_db_ = 0.9 ϵ. This particular system has shown the most efficient antidurotaxial motion (in terms of average droplet speed) among all of the cases considered in our study. (b) Side view of the same system after the droplet has moved a certain distance from its starting point. An *x* – *z* cross section passing through the center of mass of the droplet is shown to highlight the penetration of the substrate by the droplet. (c) Top view of the same configuration. (d) The distribution of the grafting sites of the brush polymer chains on the bottom solid plane of immobile beads with a honeycomb geometry is shown. At the right boundary, chains are randomly grafted in the vertical (*y*) direction with probability σ_g,h_ = 0.6 σ^–2^, while σ_g,l_ = 0.1 σ^–2^ at the left most boundary. The snapshot of the system was obtained using Ovito software.^60^

The system setup is illustrated in Figure 1. It consists of a brush substrate and a droplet. The brush has a gradient in the grafting density of the polymer chains that are tethered to a bottom wall of immobile beads with hexagonal (honeycomb) symmetry. Vertical walls of immobile beads at the two ends of the substrate that are parallel to the *y* – *z* plane with height *L*_w_ = 15 σ (σ is the length unit) are also present to support the structure of the brush in the direction of the gradient, thus extending the total area of undistorted grafting density gradient closer to the brush boundaries in the *x* direction.. The droplet is placed on the side with the highest grafting density at a distance of 30 σ between the center of mass of the droplet and the side wall, as shown in Figure 1. After examining a range of different scenarios, we have determined that in the current substrate design, the droplet motion will take place from higher grafting density areas, which implies a higher substrate stiffness toward areas of lower grafting density. In the direction of the stiffness gradient (*x* direction), the length of the substrate is *L*_*x*_ = 120 σ, while *L*_*y*_ = 60 σ is the substrate length in the *y* direction, as shown in Figure 1. Periodic boundary conditions are considered in all Cartesian directions. In particular, in the *y* direction, the boundaries of the simulation box coincide with those of the substrate, while in the *x* and *z* directions, the size of the simulation box is large enough to prevent the interaction of beads from opposite boundaries. In view of the sufficiently larger simulation box than the substrate in the direction of the gradient (*x* direction), the presence of side walls is required in order to maintain the structure of the brush in this direction.

The standard bead–spring model^61^ was employed for the molecular dynamics simulations. According to this model, the interactions between the different components of the system, i.e., the drop (*d*), the brush (*b*), and the wall (*w*) beads, are expressed through the Lennard-Jones (LJ) potential, which reads
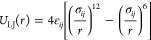
1where *r* is the distance between any pair of beads in the system. The *i* and *j* indices in eq 1 correspond to the bead type (*d*, *b*, *w*). The sizes of all of the beads are set to σ*_ij_* = σ. Moreover, the LJ potential is cut and shifted at a specific distance (cutoff), which for the interactions between the droplet beads or between the droplet and the brush polymer beads is *r*_c_ = 2.5 σ, while for all other interactions, an athermal model is used with the cutoff set to the minimum of the LJ potential, namely, *r*_c_ = 2^1/6^ σ. The potential well of the attractive interactions between the droplet beads is ε_dd_ = 1.5 ϵ, while different choices for ε_db_ are considered for the interaction strength between the droplet and the substrate.^62^ In particular, larger values of ε_db_ would correspond to fluids with a smaller surface energy, while smaller values of ε_db_ would be suitable to model fluids with larger surface energy, i.e., fluids with a lower tendency of wetting a substrate. Here, ϵ is the energy unit and the range ε_db_ = 0.1–1.2 ϵ is chosen to conduct our investigations, which allows us to capture all possible scenarios for the droplet for the specific substrate design. For all other interactions, such as those between the droplet and the walls or the brush polymers and the walls, the interaction strength is set equal to ϵ, while, as mentioned above, the model is anyway athermal for these repulsive interactions.

The size of the droplet can vary, ranging from 4 × 10^3^ to 16 × 10^3^ beads in our study. These beads are parts of fully flexible linear polymer chains. While in our investigations, the length of the droplet chains is *N*_d_ = 10 beads throughout the different simulation cases, which ensures that there are no evaporation effects and the vapor pressure is hence sufficiently low,^23,63^ chain lengths of up to 80 beads were also considered for particular cases to explore the effect of viscosity on the antidurotaxis motion.^16,23^ While the exact value of the viscosity is not of importance here and while the scaling of the viscosity with the chain length in the model seems not to have been completely settled in the literature, we can, however, note that the viscosity is expected to grow with the chain length. Moreover, a linear growth with the Kuhn length for Brownian models of melts has been observed on the basis of Rouse dynamics^64^ or a power-law relation [η] = *KM*^α^ according to Mark–Houwink–Sakurada, with *M* being the molecular weight of the polymer and *K* is determined by the intrinsic properties of the used polymer, while α = 0.8 for a good-solvent system.^65^ Hence, we expect that under melt conditions, the viscosity shall grow with a power-law exponent lower than unity for the lengths considered here, while entanglement effects are expected to also play a role for longer polymer chains. Having said that, the viscosity depends on the particular conditions (e.g., solvent conditions) when measured, but it is generally expected to increase with the chain length *N*_d_ of the chains. The length of the tethered brush polymers, which are of linear molecular architecture, is *N*_b_, and it remains the same in all of our *in silico* experiments. Based on preliminary tests, we found that this choice allows us to remove any significant dependence of the results on the choice of the brush length, *N*_b_, which is also in line with our previous experience with brush substrates.^23^

To tether the beads together in each polymer chain of the droplet or the brush, the finitely extensible nonlinear elastic (FENE) bond potential was applied for consecutive pairs of beads in each chain, which mathematically is expressed as follows
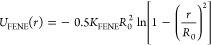
2In the above relation, *r* is the distance between the beads pair and *R*_0_ = 1.5 σ, which determines the highest possible extension of the bond. *K*_FENE_ = 30 ϵ/σ^2^ is the elastic constant.

The stiffness gradient is realized by varying the grafting density of the polymer chains in the *x* direction. By considering a hexagonal symmetry of the possible grafting sites on the bottom substrate of immobile beads, chains are randomly grafted to satisfy the required grafting density in the *x* direction (Figure 1d). In particular, the substrate is divided into bins with a length of 3 σ along the *x* direction. At one end of the substrate, we impose the highest grafting density σ_g,h_ in units of σ^–2^, while at the other end, the lowest grafting density is denoted as σ_g,l_. A linear gradient in the grafting density between the two opposite ends is considered, namely, *G* = (σ_g,h_ – σ_g,l_)/*L*_*x*_. After conducting extensive preliminary investigations with different choices for σ_g,h_ and σ_g,l_, we have concluded that setting σ_g,l_ = 0.1 σ^–2^ gives us the highest number of successful antidurotaxis cases, that is, cases that the droplet is able to cross against the gradient the whole distance from the one end of the substrate to the other. This comes as an advantage when investigating the influence of various parameters, such as the droplet size, viscosity, or adhesion strength, since more data of successful antidurotaxis cases can be acquired for the analysis. Finally, the range σ_g,h_ = 0.4–0.9 σ^–2^ was considered for the highest grafting density of the systems. Lower values of σ_g,h_ would result in small gradients, which would prevent the antidurotaxis motion. The choice σ_g,h_ ≥ 1.0 σ^–2^ already imposes large steric effects between the polymer chains and their close-packing, which would also practically imply small stiffness gradients experienced by the droplet. We have also examined systems with stiff polymer chains for the brush and concluded that antidurotaxis motion was more efficient in the case of fully flexible brush chains (no angle potential along each brush chain). This is mainly due to a smaller range of possibilities for the stiffness gradient of the substrate when individual polymer chains are stiffer. In addition, stiff chains prevent the penetration of the substrate by the droplet, which, as will become apparent later, hinders the antidurotaxis motion. Hence, all of the results presented in this study refer to brush substrates with fully flexible polymer chains.

To control the temperature of the system, *T* = ϵ/*k*_B_ (*k*_B_ is Boltzmann’s constant), a Langevin thermostat was used.^62^ Moreover, the coordinates of each bead {**r**_*i*_(*t*)} evolve in time by integrating the Langevin equation by means of the HOOMD-Blue package,^66^ which mathematically reads
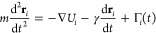
3Here, *m* is the mass of the beads, which is equal to m, with m being the mass unit; *t* denotes the time; *U_i_* is the total potential acting on the *i*th bead; γ is the friction coefficient; and Γ*_i_*(*t*) is the random force. As is well-known, γ and Γ are related by the usual fluctuation–dissipation relation
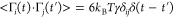
4Following previous work,^62,67,68^ the friction coefficient was chosen as γ = 0.1 τ^–1^. Equation 3 was integrated using an integration time step of Δ*t* = 0.005 τ, where the time unit is τ = (mσ^2^/ϵ)^1/2^. Given the choice of the Langevin thermostat, the simulations are, in practice, carried out in the canonical statistical ensemble. For each set of parameters, we obtained an ensemble of 13 statistically independent trajectories by changing the initial velocities assigned to each particle in order to acquire reliable statistics for the analysis of the results. The length of each trajectory was 10^8^ MD integration steps, unless the droplet managed to reach the side of the substrate with the lowest grafting density before the set maximum simulation time. In this case, the simulation is terminated, and the particular case is considered as a successful antidurotaxis case.

## Results and Discussion

In the case of the durotaxis motion onto a brush, we have determined that the key parameters of the substrate design for a droplet of a given size are the strength of droplet attraction to the substrate and the grafting density.^23^ Surprisingly, we have also found that the stiffness gradient, albeit necessary to cause the durotaxis motion of the droplet, was not per se key for determining the efficiency of the motion in terms of the time the droplet had required to transverse the full length of the substrate in the simulation. Following a similar protocol in the case of the antidurotaxis phenomenon, which includes an extensive exploration of the parameter space relevant for the new brush design, we find that the grafting density at the soft end of the substrate should be small, namely, σ_g,l_ = 0.1 σ^–2^. In addition, by investigating a relevant range of systems with different chain stiffness, we have determined that the most efficient antidutoraxis motion occurs when the brush chains are fully flexible. Henceforth, all of the presented results will consider σ_g,l_ = 0.1 σ^–2^ and fully flexible polymer chains. By taking this into account, the key parameters determining the efficiency of the antidurotaxis motion are the stiffness gradient, which is defined by the value of the grafting density at the stiffest (highest grafting density) end of the substrate with grafting density, σ_g,h_, and the droplet–brush attraction strength, which is controlled by the LJ parameter ε_db_.

**Figure 2:**
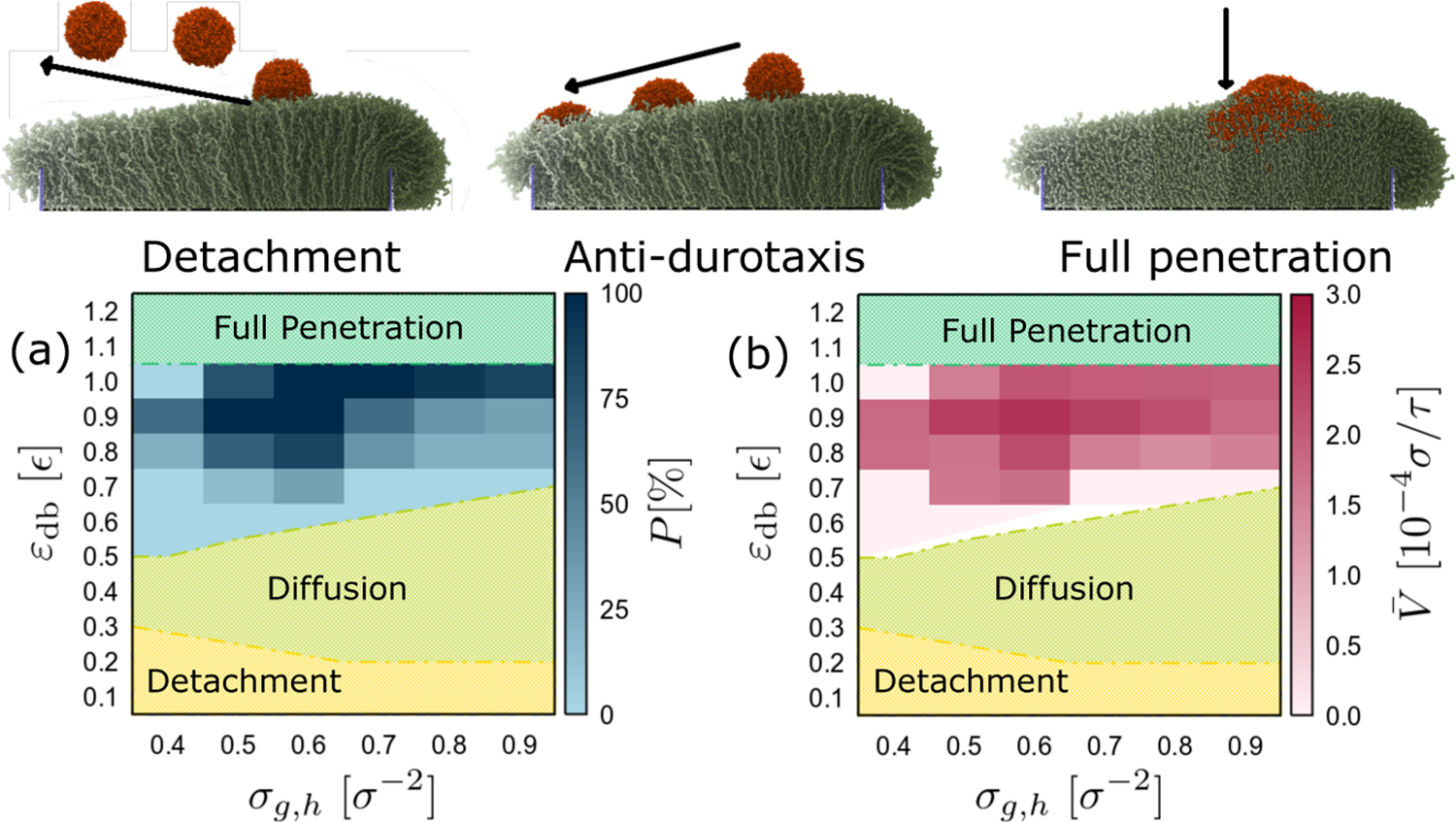
(a) Regime map indicating the probability, *P* (color scale), that a droplet will cover the full distance over the substrate in the *x* direction from the stiffest (highest grafting density) to the softest (lowest grafting density) part of the substrate (successful antidurotaxis case) for different values of the droplet–substrate attraction, ε_db_, and the highest grafting density, σ_g,h_. The lowest grafting density always has the same value, namely, σ_g,l_ = 0.1 σ^–2^. The probability, *P*, is calculated from an ensemble of 13 independent simulations for each set of parameters, ε_db_ and σ_g,h_. The regimes that the droplet immediately penetrates into the brush and is not able to further move (“full penetration”), detaches from the brush due to the weak droplet–brush attraction (“detachment”), or carries out a random walk onto the substrate (“diffusion”) are also shown with a different color. (b) The color map indicates the average velocity of the droplet, ν̅ = *L*_*x*_^′^/*t*, for the successful durotaxis cases, where *t* is the time that the droplet needs to cross the full length of the brush substrate in the *x* direction and *L*_*x*_^′^ is the actual distance covered by the center of mass of the droplet for each successful case. *N* = 4000, *N*_d_ = 10, and *N*_b_ = 50 beads. Snapshots on top of the plot indicate examples of detachment, antidurotaxis, and penetration. For the sake of providing a perspective of the various processes, in the former two cases, a time sequence of droplet snapshots at different times is shown (during this time sequence, the configuration of the brush substrate also changes with time, but a single visualization of the brush substrate is shown here only for demonstration), while in the latter case, a single snapshot of a cross-sectional view for the droplet in the brush is illustrated. The snapshot of the system was obtained using Ovito software.^60^

Figure 2 summarizes the results of the simulations in the form of regime maps with σ_g,h_ and ε_db_ as parameters. Different scenarios for the behavior of the droplet are possible during the simulations, *i.e*, its detachment from or its full penetration into the brush, a diffusion- or random-walk-like motion, and, finally, antidurotaxis motion. In particular, the detachment of the droplet takes place for a low attraction strength ε_db_. Due to the thermal fluctuations, the droplet is not able to permanently stick to the substrate, which is more probable to happen when the grafting density is lower due to a smaller number of droplet–substrate bead-pair interactions. Moreover, we observe that the dependence on the grafting density disappears beyond a certain threshold, namely, σ_g,h_ ≳ 0.6 σ^–2^, which might imply that the brush density at the interface has reached an adequately high value. In contrast, full penetration takes place when ε_db_ ≥ ϵ, irrespective of the grafting density, which suggests that full penetration be determined by the strong microscopic interactions between the substrate and the droplet beads. In this case, the droplet shape is significantly distorted by the brush chains to the extent that brush chains penetrate through the droplet chains leading to a “noncoherent” droplet (see the snapshot of Figure 2). In the third scenario, the droplet exhibits a diffusion-like (random-like) motion onto the substrate, which takes place for low ε_db_ values. This refers to unsuccessful antidurotaxis cases that are not able to cross the full length of the substrate in the *x* direction, that is in this case, the driving force of the motion is very weak. Moreover, the range of ε_db_ that such a behavior is observed increases when the grafting density σ_g,h_ increases, which already suggests that denser brushes would hinder the possibility of antidurotaxis motion. In contrast, the decreasing grafting density promotes the motion, and the specific reasons for this will become clearer later, when we discuss the underlying mechanism of the antidurotaxis phenomenon.

**Figure 3:**
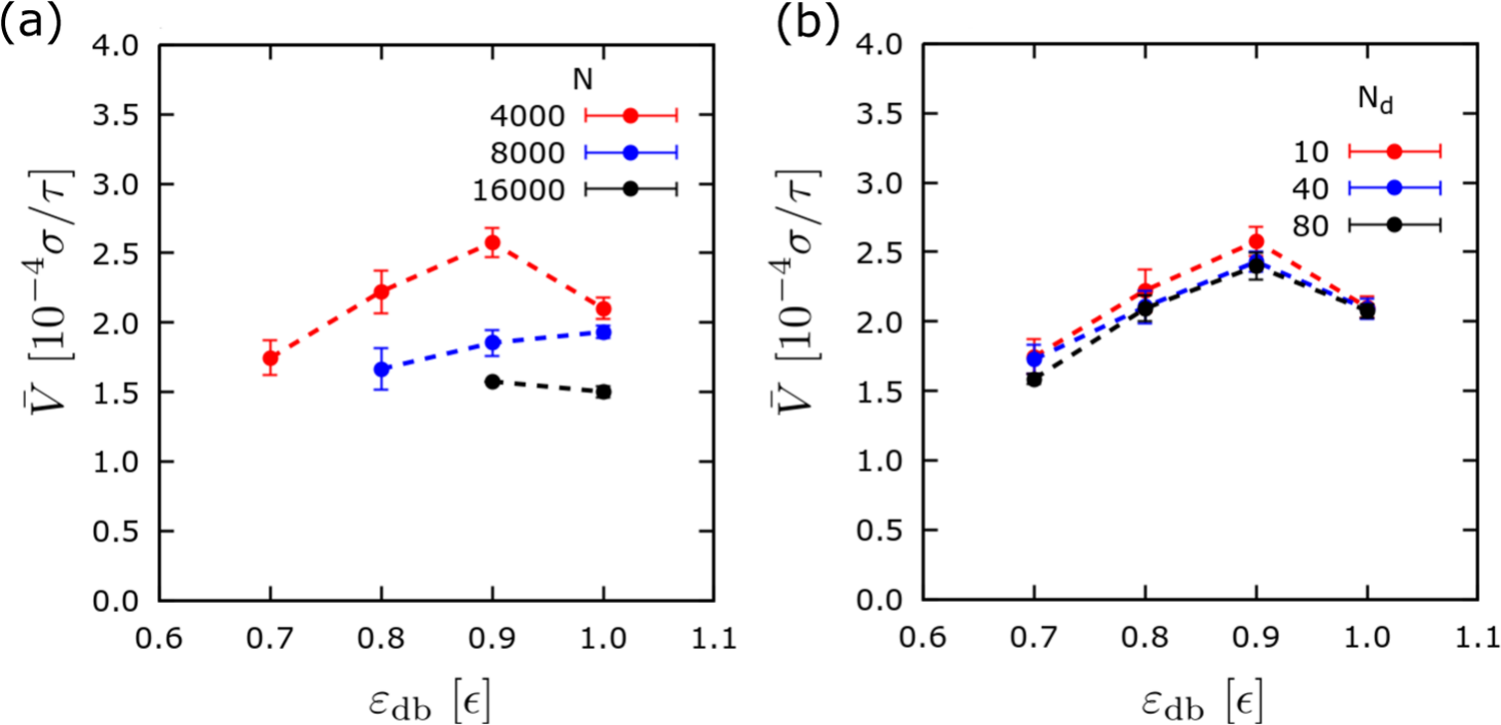
(a) Average velocity of the droplet, ν, as a function of the attraction strength, ε_db_, for successful antidurotaxis cases for different droplet size, *N*, as indicated. The average velocity of the droplet is, ν̅ = *L*_*x*_^′^/*t*, where *t* is the time that the droplet needs to cross the full length of the brush substrate in the *x* direction, and *L*_*x*_^′^ is the actual distance covered by the center of mass of the droplet for each successful antidurotaxis case. σ_g,h_ = 0.6 σ^–2^, σ_g,l_ = 0.1 σ^–2^, and *N*_d_ = 10 and *N*_b_ = 50 beads. (b) The average velocity for different chain lengths *N*_d_ of the droplet, as indicated. σ_g,h_ = 0.6 σ^–2^, σ_g,l_ = 0.1 σ^–2^, and *N*_d_ = 10, *N*_b_ = 50, and *N* = 4000 beads.

Antidurotaxis motion is observed for ε_db_ ≥ 0.7 ϵ, which suggests that a relatively high wettability of the brush by the droplet favors the motion. Moreover, we were able to find antidurotaxis for the whole range of the grafting density gradients shown in Figure 2a, but a moderate choice of the highest grafting density, in the range 0.5 σ^–2^ ≤ σ_g,h_ ≤ 0.7 σ^–2^ (the optimum choice of the grafting density on the soft end is σ_g,l_ = 0.1 σ^–2^), generally presents a high certainty for successful antidurotaxis motion with high efficiency in terms of the time required to move from the one end to the other end of the substrate. By examining more carefully this time and based on this calculation of the average velocity of the droplets during the droplet motion (Figure 2b), we find that the optimum choice is σ_g,h_ ≥ 0.6 σ^–2^ and ε_db_ = 0.9 ϵ when the droplet size is *N* = 4000 beads (Figure 3).

The results of Figure 3a indicate that increasing ε_db_ initially renders the droplet motion more efficient in terms of the average droplet velocity, but a further increase, namely, ε_db_ = ϵ, leads to a smaller velocity. The latter is attributed to the partial penetration of the brush chains into the droplet due to the strong attraction, while for ε_db_ > ϵ, we enter the “full penetration” regime (Figure 2), and the droplet motion cannot take place. As the droplet size increases, average velocities are evidently lower than those reported in the case of droplet size with *N* = 4000 beads. However, it would be difficult to identify any trends with ε_db_ for the larger droplets since the increase of the droplet size leads to a smaller number of successful antidurotaxis cases. For example, while the choice ε_db_ = 0.7 ϵ yields successful antidurotaxis motion, this choice does not show any success for droplets with the size of either *N* = 8000 or *N* = 16000 beads. A final note concerns the behavior as the droplet viscosity increases (Figure 3b). Our results indicate that its effect is rather minor since a similar behavior is observed for the droplets with chains of different *N*_d_ for different choices of attraction strength ε_db_. However, less viscous droplets (e.g., *N*_d_ = 10 beads) appear to slightly favor the antidurotaxis motion, but differences are rather within a statistical uncertainty. Finally, a decrease in the velocity for ε_db_ = ϵ is observed, which is again attributed to partial penetration of the brush chains into the droplet. Interestingly, this effect appears to be independent of the chain length and fully attributed to the microscopic interactions between the substrate and the droplet beads.

**Figure 4:**
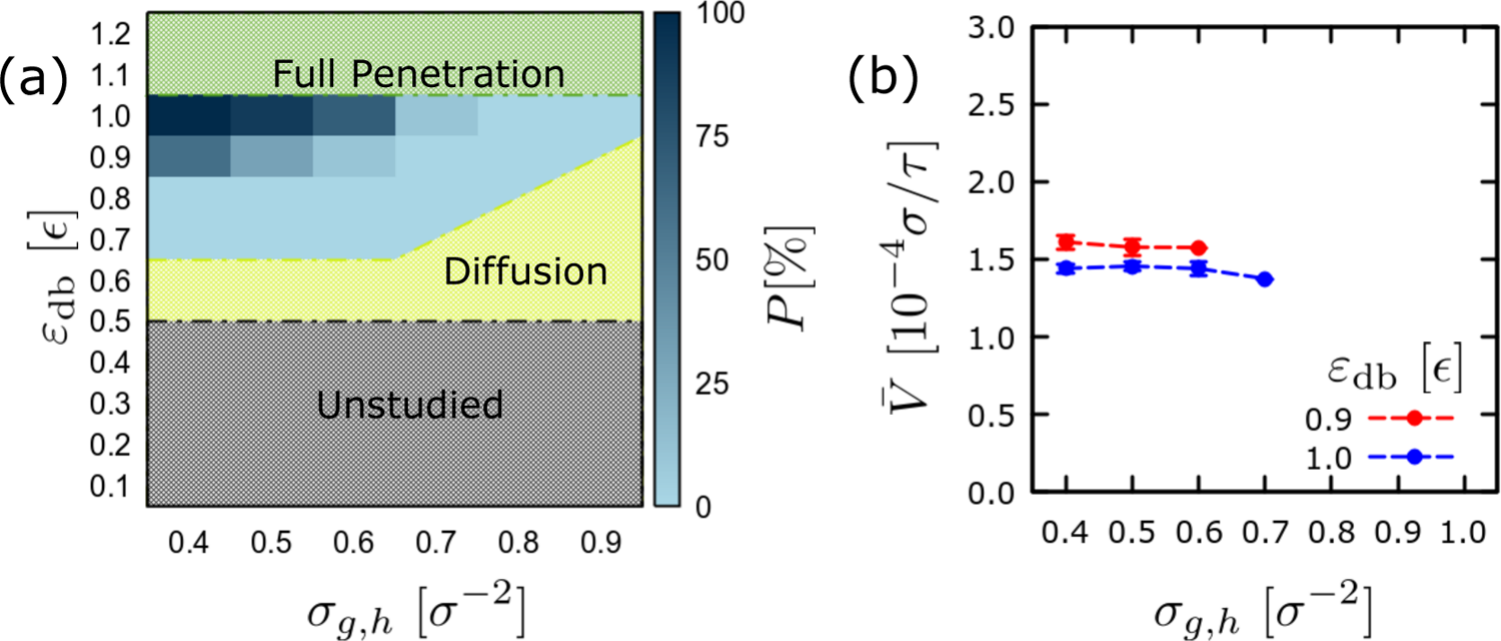
(a) Same as in Figure 2a, but results are shown for cases where the droplet consists of *N* = 16,000 beads in total. (b) Average velocity, ν, for different gradients as defined through σ_g,h_ for two different values of ε_db_ for which successful antidurotaxis motion takes place. σ_g,l_ = 0.1 σ^–2^ and *N*_d_ = 10 and *N*_b_ = 50 beads.

To carefully investigate the effect of the droplet size, we conducted extensive *in silico* experiments with a larger droplet, namely, *N* = 16,000 beads. Figure 4 presents results for this droplet size, which generally confirm the aforementioned observations. Furthermore, the regime map, which is again based on the parameters ε_db_ and σ_g,h_, shows that only a few combinations of these parameters were able to lead to successful antidurotaxis motion, and, moreover, among them, only the set of parameters ε_db_ = ϵ and σ_g,h_ = 0.4 σ^–2^ was successful in 100% of our *in silico* experiments. In addition, we observe that smaller values of σ_g,h_ favor the antidurotaxis motion in the case of the droplet with *N* = 16000 beads, in comparison with the case of *N* = 4000 beads (Figure 2). This suggests that larger droplets require a much softer substrate in order to penetrate into the brush during the antidurotaxis motion. Also, for both larger and smaller droplets, we observe that antidurotaxis is favored by larger values of ε_db_. By examining the velocity for the successful cases of ε_db_ = 0.9 ϵ and ε_db_ = ϵ (Figure 4b), we see that the average velocity remains independent of the choice of ε_db_ and that the motion is slightly more efficient when ε_db_ = 0.9 ϵ. Hence, only a narrow range of ε_db_ values can lead to successful antidurotaxis motion when the droplet size is *N* = 16,000 beads. Also, the range of σ_g,h_ for successful motion is more limited in the case of larger droplets. In summary, our data hint that more space is needed within the brush to accommodate the droplet. This will become more apparent during a detailed discussion of the motion mechanism below.

**Figure 5:**
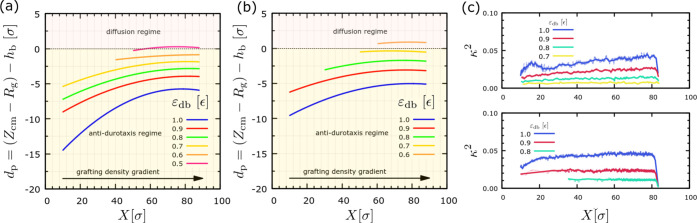
Penetration depth, *d*_p_, of the droplet into the substrate as a function of the *X* coordinate of the center of mass of the droplet along the gradient (the direction of increasing *X* is toward the regions with higher grafting density, while antidurotaxis motion is toward smaller *X* positions of the droplet) for different strength of attraction ε_db_, as indicated. Here, σ_g,h_ = 0.6 σ^–2^, σ_g,l_ = 0.1 σ^–2^, and *N*_d_ = 10 and *N*_b_ = 50 beads. Results for droplet size *N* = 4000 (a) and *N* = 16000 beads (b) are shown. (c) Dependence of the shape anisotropy parameter κ^2^ as a function of *X* for droplets with *N* = 4000 (upper panel) and *N* = 16,000 beads (lower panel). See the text for details regarding the definitions of *d*_p_ and κ^2^.

Visual observations of successful antidurotaxis cases (for example, see the snapshots in Figure 2 and Movie 1 and 2 in the Supporting Information) have led to the suspicion that the droplet gradually immerses into the brush as it moves toward the regions of lower grafting density (softer regions) since the droplet can be accommodated much easier among the brush chains. Hence, a logical next step is to attempt to characterize the degree of penetration of the droplet into the brush during the droplet motion, as part of the antidurotaxis mechanism. Moreover, it would be desirable to identify the boundaries between antidurotaxis motion and other cases, such as diffusive (nonantidurotaxis or random) droplet motion. The penetration depth serves this purpose here and is defined as *d*_p_ ≔(*Z*_cm_ – *R*_g_) – *h*_b_, where *Z*_cm_ is the center-of-mass position of the droplet in the *z* direction, *R*_g_ its radius of gyration, and *h*_b_ the height of the brush, which is identified by the inflection point of its density profile *d*ρ^2^/*dz*^2^ = 0 within the bin that the *X* position of the center of mass of the droplet belongs. Hence, the penetration depth *d*_p_ expresses the degree of the droplet immersion into the brush since *d*_p_ = −*h*_b_ when *Z*_cm_ = *R*_g_, i.e., the bottom of the droplet just touches the top of the brush. In turn, one can rescale the values of *d*_p_ according to *h*_b_, and a value of zero would simply reflect the brush surface. Figure 5 presents our results for *d*_p_ for different cases, which clearly show that the droplet penetrates deeper into the brush as it moves to areas of lower grafting density (smaller *X*) during antidurotaxis. Moreover, the droplet immersion is deeper as ε_db_ increases. This initially correlates with a higher velocity (Figure 3) of the droplet, but a further increase of ε_db_, namely, ε_db_ = ϵ, leads to a slower antidurotaxis motion, despite the difference of the average velocity between ε_db_ = 0.9 ϵ and ε_db_ = ϵ being rather small. When the size of the droplet increases (for example, see Figure 5b, where plotted data are for droplet with *N* = 16,000 beads), the conclusions remain the same, but the drop in the penetration length during antidurotaxis is much smaller than in the case of droplet with *N* = 4000 beads, which reflects a weaker effect of the gradient and a lower efficiency of motion in the case of larger droplets (for example, see Figure 3). Overall, the data indicate that successful antidurotaxis motion is strongly related to the droplet penetration into the brush.

As the droplet penetrates deeper into the brush and moves toward softer areas (smaller grafting density) in successful antidurotaxis experiments, it is relevant to investigate how the droplet shape changes during the motion. Here, we measured the shape anisotropy, κ^2^, of the droplet as a function of the position, *X* of the droplet center of mass. In particular, κ^2^ = [*b*^2^ + (3*c*^2^/4) ]/*R*_g_^4^ with , and λ_*x*_^2^, λ_*y*_^2^, and λ_*z*_^2^ are the eigenvalues of the inertia tensor for the droplet beads. *b* is the asphericity defined as λ_*z*_^2^ – (λ_*x*_^2^ + λ_*y*_^2^)/2, and *c* is the acylindricity defined as λ_*y*_^2^ – λ_*x*_^2^. The expectation is that κ^2^ obtains values closer to zero for spherical symmetry and unity in the case of a cylindrical one. The results of Figure 5c indicate that the droplet initially transforms into a quasi-spherical-cap droplet as it is deposited onto the substrate, which manifests itself by the “sharp” increase in κ^2^ and generally maintains this symmetry throughout the antidurotaxis motion with κ^2^ however remaining small throughout the motion. Also, the droplets appear to have more spherical shapes for smaller values of ε_db_. Moreover, as the droplet moves to the regions with lower grafting density, it gradually acquires a slightly more spherical symmetry due to the softness of the substrate and the more available space in these regions, despite, as we will see later, the higher number of interaction bead pairs with the surrounding brush polymer chains. Since these interactions are at the substrate–droplet interface (no full penetration as the one defined in Figure 2), they mainly minimize the energy of the system through the droplet immersion into the brush rather than leading to changes in the elastic energy of the droplet.

**Figure 6:**
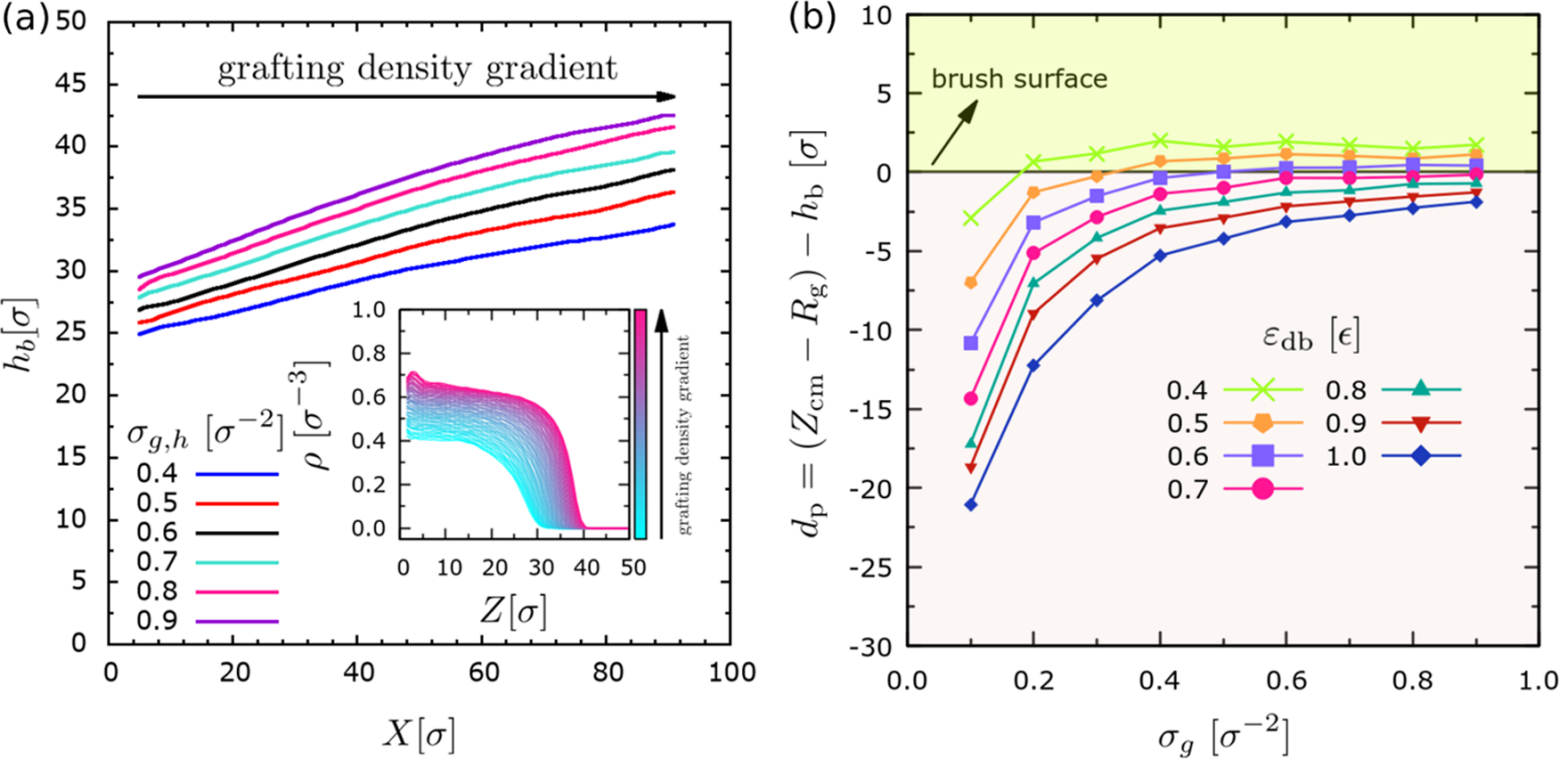
(a) Brush height, *h*_b_, calculated by the inflection point of the number-density profile, *d*ρ^2^/*dz*^2^ = 0, along the *x* direction at each position *X* for brush substrates with different gradients, as defined by different σ_g,h_. In this case, the substrate is simulated without the droplet. Also, increasing *X* values corresponds to areas with larger grafting density. Inset shows the number density of the beads, ρ, in the direction normal to the brush substrate (*z*) along the stiffness gradient (different color) for the case σ_g,h_ = 0.6 σ^–2^. (b) Depth of penetration of the droplet, *d*_p_, into substrates without gradients of different grafting densities σ_g_, as indicated. Data for different droplet–substrate attraction strengths ε_db_ are shown. In the cases of panels (a, b), data refer to cases with *N*_d_ = 10, *N* = 4000, and *N*_b_ = 50 beads.

In Figure 6a, we more closely examine the brush properties without placing the droplet on the different substrates, thus providing further evidence for our previous arguments. Our results indicate that the height of the brush, *h*_b_, decreases toward the regions with lower grafting density (smaller *X*). This trend does not depend on the particular gradient in the grafting density (different values of σ_g,h_ with σ_g,l_ = 0.1 σ^–2^ remaining constant). Hence, the slopes of the curves in Figure 6a are similar. However, larger values of the grafting density directly correlate with a larger height of the brush for a given *X* position according to the known expression *h*_b_ ∼ *N*_b_ σ_g_^1/3^.^69^ Examining the density profiles for a particular case with a specific gradient of the grafting density (inset of Figure 6a), we can also observe how the brush surface transforms from a sharper to a wider interface as we examine density profiles toward regions of lower grafting density. Hence, not only the average height, *h*_b_, of the brush changes but also the structure of the interface, which sets favorable conditions for the penetration of the droplet into the substrate. To complete the picture and better understand the above aspects, we have placed a droplet of *N* = 4000 beads onto brush substrates of different grafting density, σ_g_, and without a gradient in the grafting density of the chains and varied the strength of the attraction between the droplet and the brush (Figure 6b). Then, we measured the penetration depth, *d*_p_. First, we confirm that the droplet will penetrate deeper into the brush when the grafting density becomes smaller. From the point of view of *in silico* nanoindentation experiments, a larger penetration depth reflects a softer substrate.^70^ In the case of substrates with a larger grafting density, *d*_p_ decreases and rather reaches a plateau when σ_g,h_ > 0.4 σ^–2^. This suggests that smaller effects be expected in the droplet motion when the gradient becomes larger by setting σ_g,h_, and this might explain the lower efficiency of antidurotaxis motion for larger gradients in the grafting density (Figure 2). Second, we observe that larger droplet–substrate attraction also leads to a larger penetration depth. This effect seems to be proportional to the attraction strength for a given grafting density. Moreover, small values of ε_db_ and larger grafting density lead to situations where the droplet practically levitates on top of the brush. These cases indicate a weak attraction of the droplet to the substrate. One may assume that these will not cause antidurotaxis motion since this kind of motion is strongly driven by the interfacial interactions between the droplet and the substrate, which we will further expand upon below.

**Figure 7:**
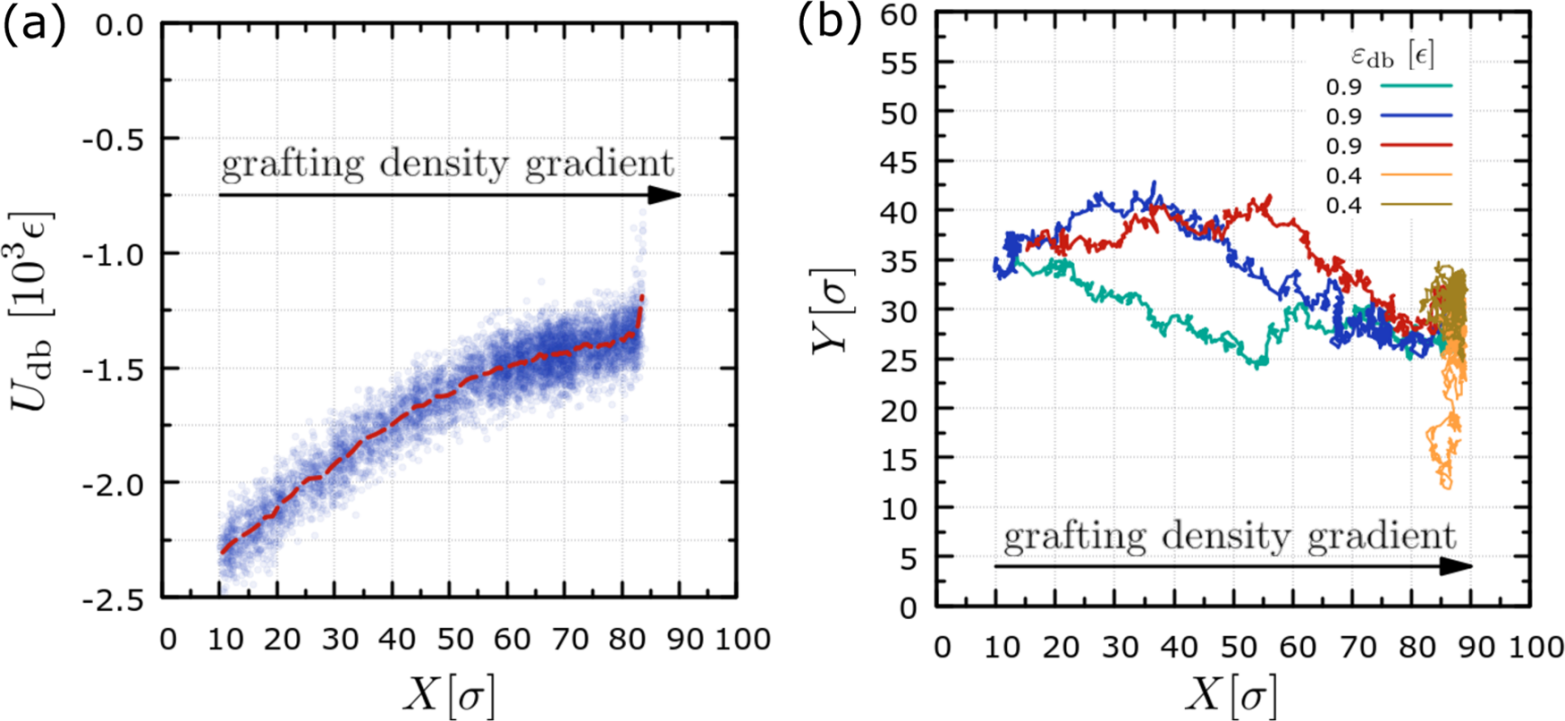
(a) Interfacial energy between the droplet and the substrate, *U*_db_, as a function of the position of the center of mass of the droplet, *X*, in the *x* direction. (b) Typical trajectories of the center-of-mass positions *X* and *Y* of the droplet on the *x* – *y* plane. Trajectories for ε_db_ = 0.9 ϵ refer to successful antidurotaxis cases with different initial conditions based on a different velocity distribution, while data for ε_db_ = 0.4 ϵ correspond to trajectories of diffusive/random droplet motion. The droplet beads in each case had different initial velocities. In both panels (a, b), *N* = 4000, *N*_d_ = 10, and *N*_b_ = 50 beads, σ_g,l_ = 0.1 σ^−2^, and σ_g,h_ = 0.6 *σ^−^*^2^, *ε*_db_ = 0.9 ϵ in panel (a).

In previous studies,^16,17,23^ it has been determined that the minimization of the interfacial energy, *U*_db_, is the driving force for durotaxis motion, which is confirmed for different substrate designs.^25^ Hence, it is also relevant for our study to examine how the droplet–brush interfacial energy varies during the antidurotaxis motion. Figure 7 presents results of the interfacial energy, *U*_db_, as a function of the center-of-mass position of the droplet, *X*, for a typical antidurotaxis case. The results confirm that the interfacial energy of the droplet decreases as it moves from the higher grafting density regions to the lower ones. As we have seen already above, this also correlates with a deeper immersion of the droplet into the brush, which indicates that the penetration of the brush by the droplet sets the conditions for energy minimization of the system. Furthermore, the energy profile is characterized by an initial smaller slope in the decrease of the energy as a function of the position *X* of the center of mass of the droplet and then by a larger slope, which correlates well with the results of the penetration depth (Figure 5), *d*_p_, and indicates that the driving force increases in the softer parts of the brush. In these softer parts, we also observe that the driving force remains constant until the completion of the antidurotaxis motion, as manifested by the constant gradient, *F*_*x*_ =- ∂ *U*_db_/∂ *x*, which is seen when approx. *X* < 60 σ. By monitoring various trajectories of successful antidurotaxis cases for a particular set of parameters (Figure 7b), we can clearly see that the motion of the droplet as viewed from the top (*x* – *y* plane of the brush) initially appears more diffusive (random) and then much more forward-moving, thus closely reflecting the observations regarding the driving force in Figure 7a. For the sake of comparison, we also show cases of unsuccessful antidurotaxis attempts for low ε_db_ values, where droplets show a random motion around the very initial position *X* of the droplet on the substrate. This further indicates that antidurotaxis motion is not governed by random fluctuations, but it is a result of the particular choice of a set of parameters leading to a driving force, *F*_*x*_, as in previous *in silico* experiments.^23^ In our case, the key set of parameters is specifically the gradient in the grafting density given the choice σ_g,l_ = 0.1 σ^–2^ and the choice of materials for the brush and the droplet, which will eventually determine the strength of the interaction between these two system components.

**Figure 8:**
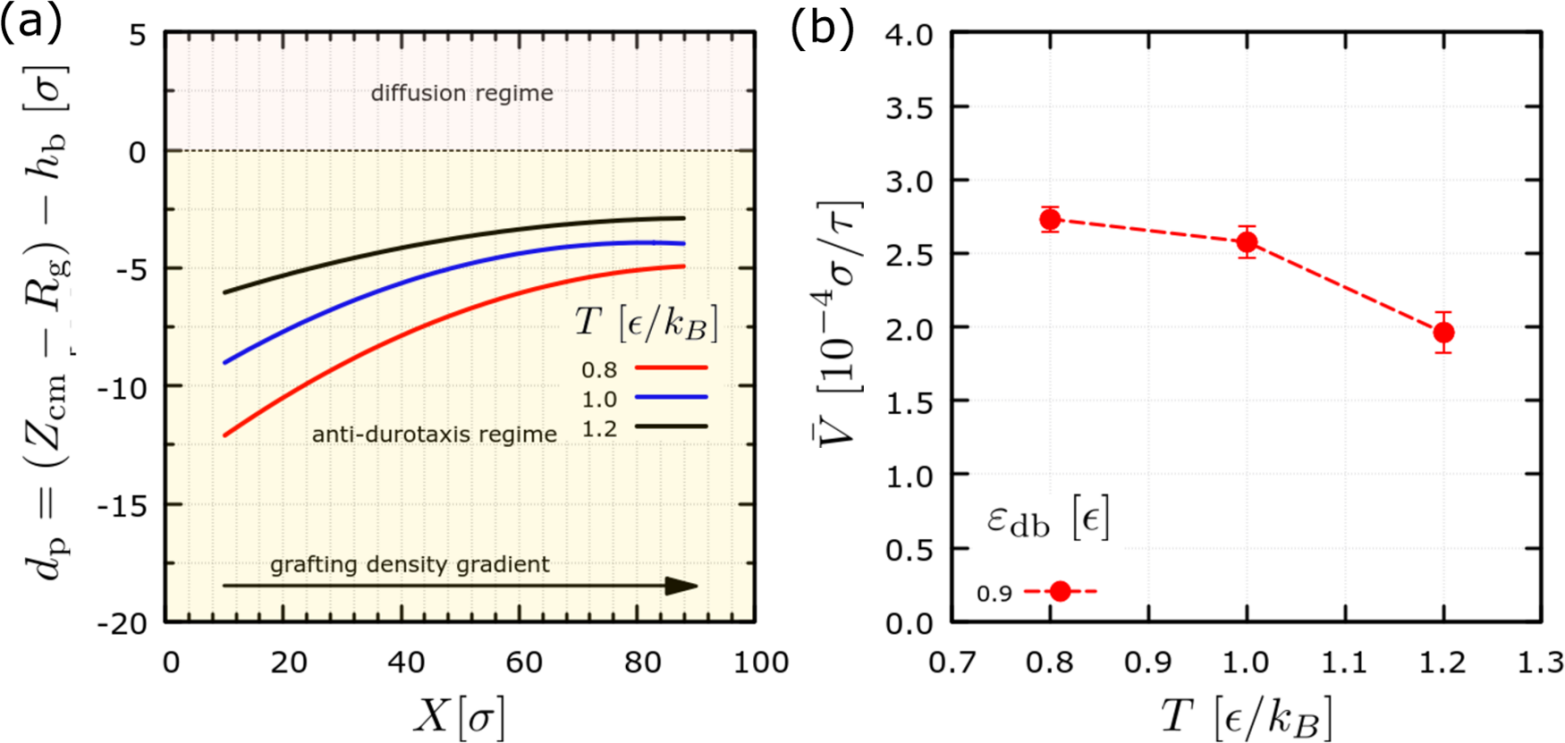
(a) Penetration depth, *d*_p_, as a function of the position, *X*, of the center of mass of the droplet for different system temperatures, *T*, as indicated. (b) Average velocity of the droplet as a function of the temperature. Here, *N* = 4000, *N*_d_ = 10, and *N*_b_ = 50 beads. ε_db_ = 0.9 ϵ and σ_g,h_ = 0.6 σ^–2^. Data are based on an ensemble of 13 independent trajectories for each set of parameters to obtain sufficient statistics.

In the following, we explore the effect of the system temperature on the antidurotaxis motion. A case of highest motion efficiency is chosen to facilitate the analysis, and the temperature of the system is varied. Overall, we find that antidurotaxis motion will take place for all of the three temperatures studied here (one temperature lower and another greater than ϵ/*k*_*B*_). Moreover, we monitor the penetration depth and calculate the average velocity of the droplet center of mass, with the results presented in Figure 8. We find that the motion becomes more efficient (on average, the droplet moves faster) in the case of the lowest temperature (Figure 8b). Moreover, we find that the more efficient motion correlates well with a larger penetration depth of the droplet and a larger slope of the depth as the droplet moves toward regions with smaller grafting density (Figure 8a). Hence, we may argue that the antidurotaxis motion be more efficient at lower temperatures, when thermal fluctuations are less pronounced in the system, and crucial at the droplet–substrate interface, which provides further evidence on the underlying mechanism of the droplet motion, that is, the minimization of the droplet–substrate interfacial energy as the droplet is able to establish a larger number of interaction contacts with the brush during antidurotaxis motion. Note that the brush chains do not penetrate into the droplet chains, and therefore, antidutoraxis is fully controlled by the interfacial interactions, as in the case of another brush-substrate design.^23^ The increase in the temperature generally leads to a decrease in the surface tension of the droplet. However, temperature is also observed to affect the substrate properties. Hence, the synergistic effect of the temperature can only accurately be assessed by simulating systems at different temperatures, as is done in our study.

**Figure 9:**
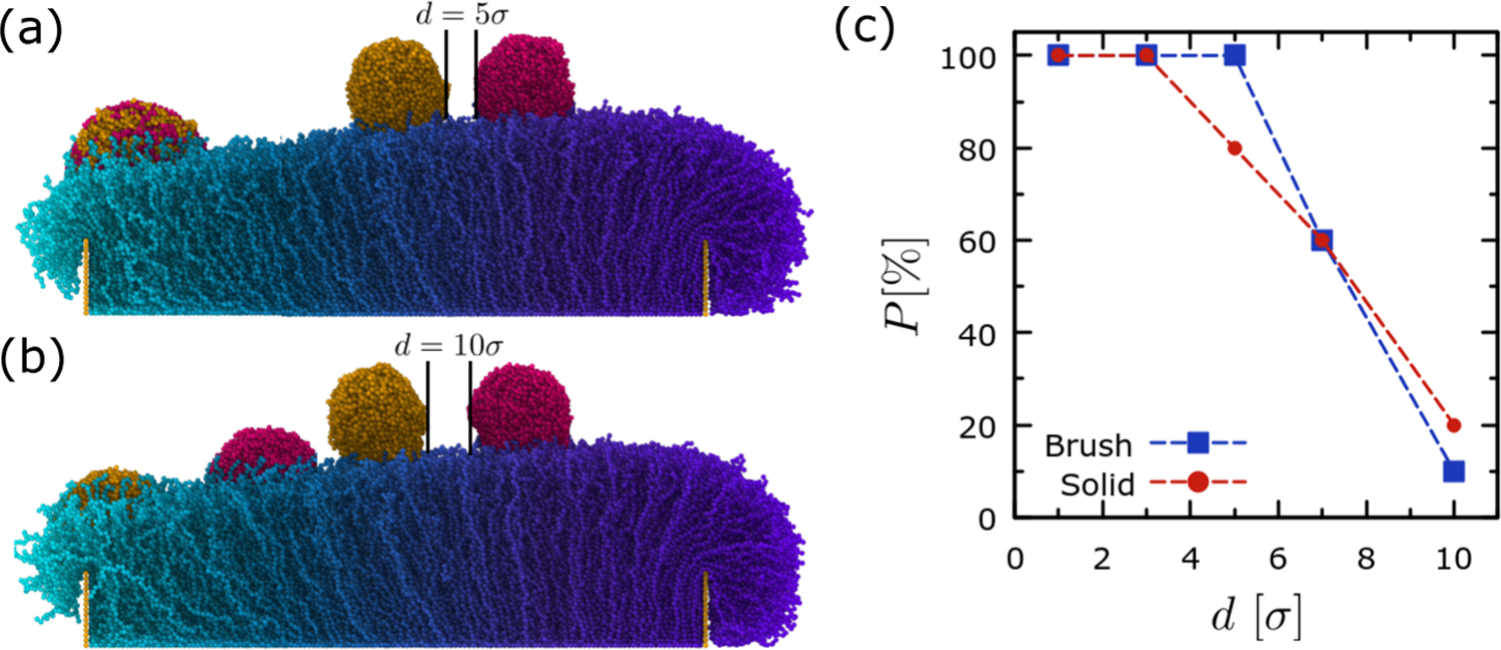
Antidurotaxis motion of two droplets, which are initially placed at a closer distance (i.e, 5 σ) and coalescence (a) and at a larger distance (i.e., 10 σ) avoiding coalescence (b). In panels (a, b), the same snapshot has been used in each case since the substrate is only used for visualization purposes (see Movies 3 and 4 in Supporting Information for more representative examples.) In both panels (a, b), the snapshots of the droplets are taken at an initial time and at a later time when both (a) or at least one (b) droplet has reached the substrate end with the lowest grafting density. (c) Probability that droplets coalesce as a function of the initial distance between the droplets, *d*. An ensemble of 10 independent trajectories has been considered for the analysis. Here, *N*_d_ = 10, *N*_b_ = 50, and *N* = 4000 beads. ε_db_ = 0.9 ϵ, σ_g,h_ = 0.6 σ^–2^, and σ_g,l_ = 0.1 σ^–2^. The different color of the droplets is only to distinguish them visually. The snapshot of the system was obtained using Ovito software.^60^

Although the study of a single droplet on gradient substrates offers advantages for *in silico* experiments, for example, isolating the various effects and carefully investigating the droplet–substrate interactions, multiple droplets on the same substrate are often used to carry out the studies in real experiments.^24^ This might be of benefit, for example, in gathering statistics over a larger number of droplets within an individual experiment. While the focus of this study is on the antidurotaxis motion of a single droplet, we also performed *in silico* experiments with two droplets and carried out an ensemble of 10 trajectories for each case to explore the behavior of the system in such a scenario. Again, the most efficient case was chosen for the investigations, with results presented in Figure 9. Here, the main focus is placed on the role of the substrate in the coalescence of the droplets, in other words to probe whether the brush would favor the droplet coalescence by acting as a “bridge” between the droplets, given the softness of the substrate, or the brush chains would rather act as a “barrier” that prevents the coalescence of the droplets. To answer this question, we place two droplets at different distances between each other onto two different substrates, namely, the brush substrate with a gradient (Figure 9) and a smooth, unstructured substrate without a gradient modeled by a 12–6 LJ potential assuming the same set of interaction parameters. We find that the droplets will first coalescence and then move together as a larger droplet due to antidurotaxis when the distance is small enough in the case of the brush substrate. Moreover, the probability of coalescence depends on the distance *d* between the droplets, with the brush substrate favoring coalescence over slightly larger distances in comparison with the case where the droplets are placed on the solid substrate. In particular, for *d* ≤ 3 σ, both substrates will lead to droplet coalescence with 100% probability. The solid substrate then will provide droplet coalescence with a smaller probability, and after a distance *d* ≥ 6 σ, the probability is more or less the same for both substrate types. Since the potential cutoff is 2.5 σ, this might suggest that the solid, smooth, unstructured substrate does not favor coalescence when the droplets are not able to “feel” each other. In contrast, the brush substrate gives a 100% probability of coalescence even when the distance between the droplets is 5 σ, which is twice the cutoff distance of the potential. We might argue that the brush chains in this case fill in the space between the droplets and act in favor of coalescence. A further increase of the initial distance between the droplets, *d*, however, will weaken this effect, and the behavior of the droplets in terms of coalescence is the same for both the solid and the brush substrate. Finally, we find that when coalescence between the droplets is avoided, it will not take place later during the antidurotaxis motion of the droplets (Figure 9b, see also Movie 3 and 4 in Supporting Information for the coalescence and noncoalescence cases of Figure 9). It would be interesting to explore systems with multiple droplets in the future and attempt to further estimate the role of the brush chains as mediators between the droplets or explore synergistic effects that might be relevant in the antidurotaxis motion in systems of multiple and diverse droplets, but this clearly goes beyond the scope of the current study.

## Conclusions

In this study, we proposed and investigated a new design of a brush substrate that is able to lead to the self-sustained motion of droplets without an external energy source. An important difference of this substrate is that the droplet motion takes place in the opposite direction of the stiffness gradient, hence the term antidurotaxis that is coined in our work. As such, to the best of our knowledge, this is the first time that antidurotaxis motion has been observed in simulations since previous cases were mainly concerned with durotaxis motion, that is, droplet motion in the direction of the stiffness gradient from softer to stiffer regions of a substrate.^16,17,23^ Also, durotaxis motion is usually observed in experiments in the lab and biological systems,^11−15^ while antidurotaxis motion has only been observed for a particular experimental setup.^24^

As in the case of durotaxis onto brush substrates,^23^ our analysis here confirms that the minimization of the interfacial energy constitutes the driving force that underpins the antidurotaxis phenomenon. However, in the case of antidurotaxis, this minimization is caused by the gradual penetration of the droplet into the brush as the droplet moves to the softer parts of the substrate with lower grafting density. We also conducted a parametric study in order to gain further insights into the influence of the various system parameters. We have concluded that soft brushes are in general more suitable for antidurotaxis; therefore, fully flexible polymer chains and the lowest grafting density on the one end of the substrate were always kept σ_g,l_ = 0.1 σ^–2^. Then, the two key parameters defining the probability of success and the efficiency of the motion in terms of the average droplet velocity are the gradient in the grafting density and the droplet–brush attraction strength. In particular, we find that large values of the droplet–brush interaction strength (low surface energy) favor the antidurotaxis motion, when full penetration of the droplet is avoided, namely, ε_db_ = 0.9 ϵ approx. Then, moderate values of the grafting density gradient are preferable, in particular, σ_g,h_ = 0.6 σ^–2^. This is due to the requirement of maintaining the substrate softness that allows its penetration by the droplet while maintaining the highest possible gradient, i.e., the highest possible driving force. As the droplet size increases, smaller gradients perform better, but the antidurotaxis motion overall is less efficient in the case of larger droplets. The temperature also plays an important role. We find that a lower temperature will favor the antidurotaxis motion, which provides further evidence supporting the minimization of the droplet–substrate interfacial energy as the driving force of antidurotaxis. Finally, we find that the viscosity has a minor effect on the antidurotaxis droplet motion, as it has been also observed in the case of the durotaxis motion for a different brush-substrate design.^23^

The key element of the new brush design is the gradient in the grafting density, and therefore, it might be more applicable in the case of experiments since the chemically same type of monomers can be used for all chains. Hence, our findings might motivate further experimental research in the area of self-sustained fluid motion on brush gradient substrates. This might be combined with experiments that include the population of droplets, which may provide further understanding of the underlying mechanisms of antidurotaxis. Here, we have also found that brush substrates favor the coalescence of droplets placed at a close distance in comparison with smooth, unstructured, solid substrates. Another aspect that may require further investigation in the future is to assess possible effects of capillary wavelike fluctuations along the brush surface by simulating much larger systems. We expect that future studies will provide experimental evidence of the plethora of possibilities unfolding regarding antidurotaxis motion. Also, we anticipate that this study already highlights new possibilities in the design of gradient substrates and, as the first antidurotaxis *in silico* design, holds important implications for various technological areas, as it aims at forging understanding of the underlying mechanisms that underpin this and similar phenomena.

## Supporting Information Available

Movie illustrates an example of antidurotaxis motion onto a gradient brush substrate, and the parameters for the system are *N* = 4000, *N*_d_ = 10, and *N*_b_ = 50 beads, σ_g,h_ = 0.6 σ^–2^, σ_g,l_ = 0.1 σ^–2^, and ε_db_ = 0.9 ϵ (Movie 1) (MP4)
Movie illustrates a different view of the system in Movie 1 (Movie 2) (MP4)
Movie illustrates the case of Figure 9 (Movie 3) (MP4)
Movie illustrates the case of Figure 9b (Movie 4) (MP4)
